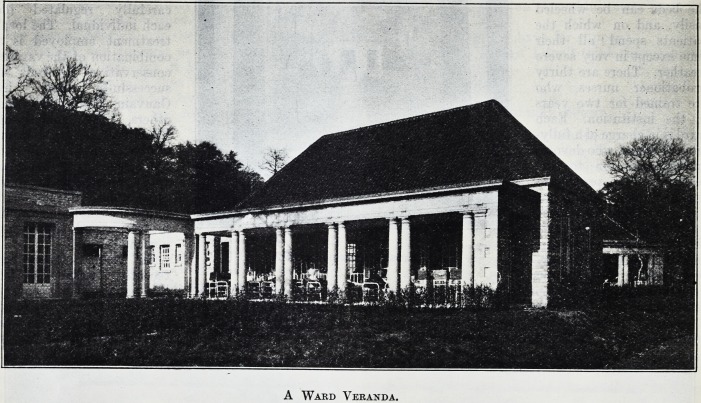# The Tuberculous Child

**Published:** 1924-09

**Authors:** C. Lee Pattison

**Affiliations:** Surgical Tuberculosis Officer to the City of Sheffield


### THE TUBERCULOUS CHILD.

### SHEFFIELD'S LITTLE CRIPPLES.

By C. LEE PATTISON, M.B., B.S., M R.C.S., L.R.C.P., Surgical Tuberculosis Officer to the City of Sheffield.

The King Edward VII. Hospital exists for the treatment of Sheffield children under fourteen years of age—who are suffering with tuberculosis of bones or joints. It is beautifully situated in a wood on the northern side of the Rivelin Valley, about five miles from Sheffield. The administrative block and nurses' home is in the centre, and the six wards (each of twenty beds) are placed to the east and west of this, being joined to it by a covered corridor. The wards open upon wide verandas to which the beds can be wheeled easily, and on which the patients spend all their time except in very severe weather. There are thirty probationer nurses, who are trained for two years in the institution. Each ward is in charge of a fully-trained Sister, who devotes special attention to the teaching of the probationers. Lectures are also given, and a certificate is granted at the end of two years to those who pass an examination. Arrangements have been made with various general hospitals to take probationers who have done satisfactory work, and to " let them off " one year's " general " training. Each nurse has a separate bedroom, and a hockey field and tennis court afford the sisters and nurses recreation and exercise, which has been found of great value in increasing the happiness and health of the staff.

A Kiosk in the Grounds.

The hospital contains an operating theatre, plaster room, splint room, pathological laboratory and a modern X-ray installation. Facilities for treatment with ultra-violet radiations from various types of lamp are also provided. Whenever it is warm enough, the patients are exposed to air and light on the ward verandas, the degree of exposure being carefully regulated for each individual. The local treatment employed is a combination of the various conservative methods so successfully used by Gauvain, Girdlestone and others, the underlying principles being fixation of the diseased joint and prevention of muscular spasm, care being taken to assist the development round the lesion of a strong protective and supporting musculature.

Sir Robert Jones has recently written :—" To start a (crippled) child on the right road to recovery is not sufficient ; if the goal is to be reached he must be carefully steered by efficient after-care." It was very early recognised in Sheffield that an institution

The Entrance Hall.

like the King Edward VII. Hospital only provided part of the necessary mechanism for dealing with tuberculous cripples; that in a long disease such as osseous tuberculosis it was only possible to treat patients with actively progressing and acute disease in hospital; and that it was essential to provide very thorough arrangements for supervision when the patients returned home, both to see that the instructions for the care of the children were satisfactory and were being carried out, and so that patients could be readmitted immediately should they show any recurrence of active disease. It is beyond the scope of this article to describe the arrangements for out-patients; it is sufficient to say that an adequate system of " after-care " has been developed, which is proving satisfactory. There are over 1,000 tuberculous cripples in Sheffield who are in various stages of convalescence, and about 150 fresh cases are seen every year, so that the scope of the work is rapidly increasing.

A Ward Veranda.

### HOW INSULIN ACTS.

We have already become so accustomed to the virtues and benefits of insulin that it may come as a surprise to realise that we do not yet know how this substance acts. The workers associated with Prof. J. J. R. Macleod are still activeiy engaged upon the problem. The point is that if sugar and insulin are added to fresh blood or tissues *in vitro*, the sugar is not used up any faster than it would be if no insulin were there. The substance injected into living tissues accelerates the rate of sugar absorption. The problem to be solved is whether insulin acts in the living blood stream only after it has there combined with some other active principle or enzyme, or whether it first passes into the living cells of the body and is in them activated by some unknown substance.

## Figures and Tables

**Figure f1:**
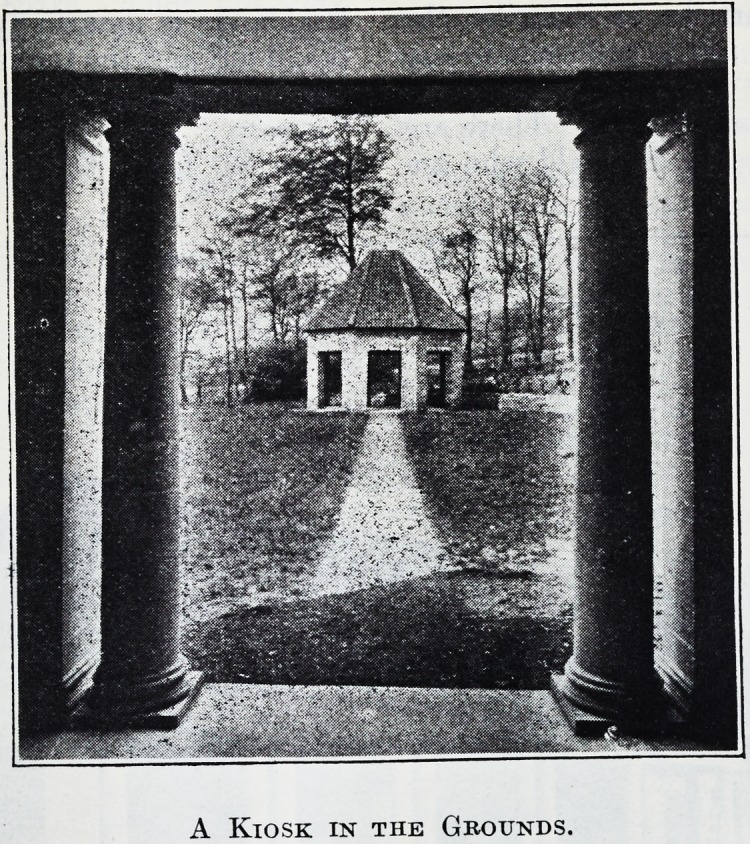


**Figure f2:**
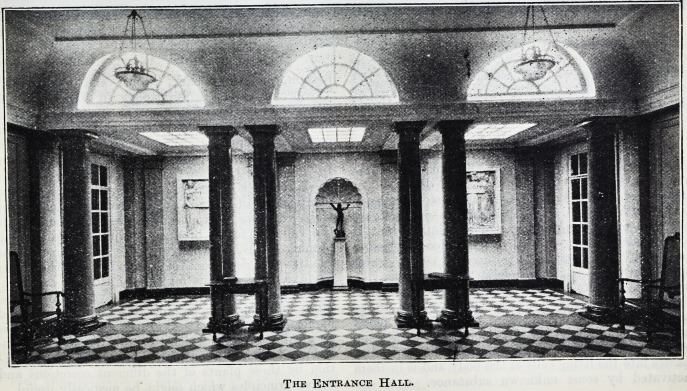


**Figure f3:**